# RFRP-3, the Mammalian Ortholog of GnIH, Is a Novel Modulator Involved in Food Intake and Glucose Homeostasis

**DOI:** 10.3389/fendo.2020.00194

**Published:** 2020-04-09

**Authors:** Konglin Huo, Xun Li, Wen Hu, Xingxing Song, Duoni Zhang, Xin Zhang, Xinyi Chen, Jingzhi Yuan, Jianyu Zuo, Xiaoye Wang

**Affiliations:** College of Animal Science and Technology, Guangxi University, Nanning, China

**Keywords:** RFamide-related peptide-3 (RFRP-3), food intake, insulin, glucagon, insulin resistance, glucose metabolism

## Abstract

RF amide-related peptide 3 (RFRP-3) is a reproductive inhibitor and an endogenous orexigenic neuropeptide that may be involved in energy homeostasis. In this study, we evaluated the effect of acute or chronic RFRP-3 treatment (administered via intraperitoneal injection) on the food intake, meal microstructure and weight of rats, as well as the mechanism through which RFRP-3 is involved in glucose metabolism in the pancreas and glucose disposal tissues of rat *in vivo*. Our results showed that the intraperitoneal administration of RFRP-3 to rats resulted in marked body mass increased, hyperphagia, hyperlipidemia, hyperglycemia, glucose intolerance, hypoinsulinism, hyperglucagon, and insulin resistance, as well as significant increases in the size of pancreatic islets and the inflammatory reaction. Thus, we strongly assert that RFRP-3 as a novel neuroendocrine regulator involved in blood glucose homeostasis.

## Introduction

Gonadotropin-inhibitory hormone (GnIH) was the first avian RFamide peptide identified that directly acts on the pituitary to inhibit gonadotropin release from the quail hypothalamus (1). After the discovery of GnIH in birds, GnIH orthologs, known as RFamide-related peptide-3 (RFRP-3), were subsequently identified in a number of mammals (2, 3). After nearly 20 years of research, it has been established that RFRP-3 plays a role in regulating mammalian reproduction through the hypothalamic-pituitary-gonadal (HPG) axis via its G protein-coupled receptor 147 (GPR147) (4, 5).

Reproduction is highly sensitive to changes in the metabolic status and energy reserves of an organism, and adverse metabolic conditions are commonly associated with defective reproductive capacity (6). In this context, studies focusing on the reproductive functions of the RFRP-3 system have also noted a potential role for RFRP-3 in appetite regulation (7, 8). Exogenous administration of GnIH/RFRP-3 was shown to potently stimulate food intake in chickens and rats (9, 10). This evidence for a regulatory role of RFRP-3 in reproduction and appetite suggests that this neuropeptide may be important for the integration of energy balance and reproductive function. In addition, because of its ability to inhibit reproductive function and to stimulate food intake, RFRP-3 may contribute to a negative energy balance. However, unequivocal evidence for such a role is largely speculative, and the physiological mechanisms by which RFRP-3 affects energy homeostasis remain unknown. Thus, the aim of the present study was to investigate the effect of RFRP-3 on food intake and glucose metabolic regulation in rats.

## Materials and Methods

### Animals and RFRP-3

All of the experiments were performed in accordance with the guidelines of the regional Animal Ethics Committee and were approved by the Guangxi University Ethical Committee (Project ID 2019-068). Sprague-Dawley rats (6 weeks old, 220 ± 10 g) were obtained from the inbred colony maintained in the Guangxi Medical University of Laboratory Animals Center. The animals were maintained under constant conditions of light (12:12 light-dark cycle) and temperature (25°C) with free access to rodent food (65% carbohydrate, 25% protein, and 10% fat) and water. Rats of both sexes were used for all experiments, as no differences between the sexes were observed. Thus, all data from this study include both sexes.

RFRP-3 (catalog No. 048-52, Phoenix Pharmaceuticals, USA) was used in the present study.

### Food Intake, Meal Microstructure, and Weight Measurements

Rats (*n* = 10 per group, half male and half female) received chronic intraperitoneal injections of different doses of RFRP-3 (0, 1, and 10 μg/100 μL, total 200 μL of RFRP-3 dissolved in a 0.9% saline solution) twice a day (7 a.m. and 7 p.m.) for 14 days. Meal microstructure monitoring was conducted as detailed in a previous study (11). After 14 days, the rats were weighed and then sacrificed by decapitation 15 min later after the last injection to measure serum total triglyceride and cholesterol levels using an automatic biochemical analyzer (URIT-8021AVeT, Uritest, CN). In addition, the tissues were harvested and stored at −80°C until further processed.

### Blood Glucose and Serum Glucometabolism-Related Hormone Measurements

Rats (*n* = 10 per group, half male and half female) that had fasted for 8 h received acute or chronic intraperitoneal injections of different doses of RFRP-3 as described above. Blood glucose was immediately measured using a blood glucose meter (FreeStyle Optium Neo, Abbott, CN) from tail vein blood samples at specific time points (15 min prior to and 0–120 min after an injection of RFRP-3). Fifteen minutes after injection, serum insulin, glucagon, epinephrine, leptin, and growth hormone levels were assessed by enzyme-linked immunosorbent assay (catalog No. CEA448Ra, CEB266Ra, CEA858Ge, SEA084Ra, and SEA044Ra; Cloud-Clone Corp, CN). The enzyme-linked immunosorbent assay sensitivity of insulin, glucagon, epinephrine, leptin and growth hormone was 50.2, 7.44, 8.92 pg/ml, 0.129 and 0.055 ng/ml, separately.

### Glucose Tolerance Test

Before glucose tolerance test, chronic RFRP-3-injected rats received intraperitoneal injections of different doses of RFRP-3 twice a day for 14 days as described above, whereas the rats of acute treatment group were reared without any treatment. After fasting for 8 h, rats in acute and chronic treatment groups were all intraperitoneally injected with glucose (Kelun Medicine, Hubei, CN) at a dose of 2 g/kg body weight. 15 min later, different doses of RFRP-3 were intraperitoneally injected into the rats of all groups. Subsequently, blood glucose was immediately measured from tail vein blood samples at specific time points as described above.

### Insulin Tolerance Test

Before insulin tolerance test, chronic RFRP-3-injected rats received intraperitoneal injections of different doses of RFRP-3 twice a day for 14 days as described above, whereas the rats of acute treatment group were reared without any treatment. After fasting for 8 h, rats in acute and chronic treatment groups were all intraperitoneally injected with insulin (Wanbang, Jiangsu, CN) at a dose of 0.5 IU/kg body weight. 15 min later, different doses of RFRP-3 were intraperitoneally injected into the rats of all groups. Subsequently, blood glucose was immediately measured from tail vein blood samples at specific time points as described above.

### Gene Expression

The pancreas, liver, muscle, white adipose tissue, and hypothalamus were excised from the control rats or rats having undergone chronic RFRP-3 intraperitoneal administration for 14 days. Relative real-time RT-PCR and semi-quantitative RT-PCR were performed as described in our previous study (12, 13). Amplification reactions were conducted in triplicate using gene-specific primers designed from the clone sequences shown in Table 1. The quantitative RT-PCR was performed using 2^−ΔΔCT^ method with GAPDH as the internal control for normalization.

**Table 1:** Primers and annealing temperature for relative real-time RT-PCR and semi-quantitative RT-PCR.

**Genes**	**Primer sequence (5'-3')**	**Annealing (^**°**^C)**
Insulin	F:ATTGTTCCAACATGGCCCTGT	61
	R:TTGCAGTAGTTCTCCAGTT	
Glucagon	F:ACGAATACATTTCCCTTTAGCG	60
	R:TGTTGTTGTAATCCAGGTGTCG	
GLUT4	F:CTCTCAGGCATCAATGCTGTT	60
	R:GAGACCAACGTGAAGACGGTA	
Insulin receptor	F:CTGGAGAACTGCTCGGTCATT	60
	R:GGCCATAGACACGGAAAAGAAG	
IL-1β	F:TGAGGCTGACAGACCCCAAAAGAT	60
	R:GCTCCACGGGCAAGACATAGGTAG	
TNF-α	F:CACGTCGTAGCAAACCACCAA	60
	R:GTTGGTTGTCTTTGAGATCCAT	
β-actin	F:ACTTCGAGCAGGAGATGGCC	60
	R:CCCAAGAAGGAAGGCTGGAA	
GPR147	F:AGCCTCACCTTCTCCTCCTACTACC	60
	R:AGTGATAAGGTTGTCCACAAGGGTT	
RFRP-3	F:GAGGAATCCCAAAAGGGGTAAAGG	60
	R:GTGATGCGTCTGGCTGTTGTTCT	
GAPDH	F:ACCACAGTCCATGCCATCAC	55
	R:TCCACCACCCTGTTGCTGTA	

### Western Blot Analysis

The pancreas, liver, skeletal muscle, white adipose tissue, and hypothalamus were excised from the control rats or rats having undergone chronic RFRP-3 intraperitoneal administration for 14 days and lysed in a cell lysis buffer (Beyotime) containing 1 mM phenylmethylsulfonyl fluoride. Western blotting was conducted as described in our previous study (13). Blotted membranes were incubated with primary antibodies at the appropriate dilution [GSK-3β (1:3000 dilution, Cell Signaling Technology), Phospho-GSK-3β, AKT, Phospho-AKT, GAPDH (1:2000 dilution, Cell Signaling Technology), GPR147 (1:2000 dilution, Biorbyt), and RFRP-3 (1:1000 dilution, Biorbyt Ltd., UK)] and were then incubated with horseradish peroxidase-labeled goat anti-rabbit or anti-mouse IgG secondary antibody (1:20,000 dilution, Cell Signaling Technology). Densitometric quantification was performed with ImageJ using GAPDH as the internal control for normalization.

### Morphological Analysis

The cauda pancreas tissues from the control rats or rats having undergone chronic RFRP-3 intraperitoneal administration for 14 days were excised and fixed in neutral-buffered formalin. Tissue samples were dehydrated in a graded ethanol series and embedded in paraffin wax followed by histological sectioning (5 mm). The antibody specificity for immunohistochemistry and immunofluorescence were conducted as described in our previously study (13, 14). In the immunohistochemistry and immunofluorescence analysis, the tissue sections were incubated with a primary antibody at the appropriate dilution [insulin and glucagon (1:100 dilution, Boster Biological Technology), GPR147 (1:250 dilution, Biorbyt), and RFRP-3 (as described previously)]. Immunohistochemical analyses were performed using streptavidin-biotin-peroxidase complex (SABC) kits (catalog No. SA1021 and SA1022, Boster Biological Technology). After primary antibody incubation, the sections were incubated with Cy3-conjugated goat anti-rabbit IgG or DyLight 488-conjugated goat anti-mouse IgG secondary antibodies (1:200 dilution, Boster Biological Technology). 4',6-Diamidino-2-phenylindole (Solarbio) was used to counterstain the nuclei, and the microscopic images were recorded by laser confocal microscopy (TCS sp8 Leica) or fluorescence microscopy (Nikon). The parameters for the pancreatic islet cytoarchitecture were analyzed as described in a previous study (15, 16).

### Protein Chip Assay

Protein chips were obtained from RayBiotech [A Quantibody Rat Inflammation Array 1 (QAR-INF-1-4, RayBiotech, Inc., Norcross, GA)], and experiments were performed as described previously (17).

### Statistical Analyses

The statistical analysis was evaluated by unpaired two-tailed Student's *t*-test or one-way ANOVA with SPSS Statistics version 17.0. The differences were considered to be significant when *P* < 0.05. No data were excluded from the analyses.

## Results

### Intraperitoneally Injected RFRP-3 Increases Rat Obesity and Photophase Food Intake and Alters Meal Microstructure

**Figure 1:**
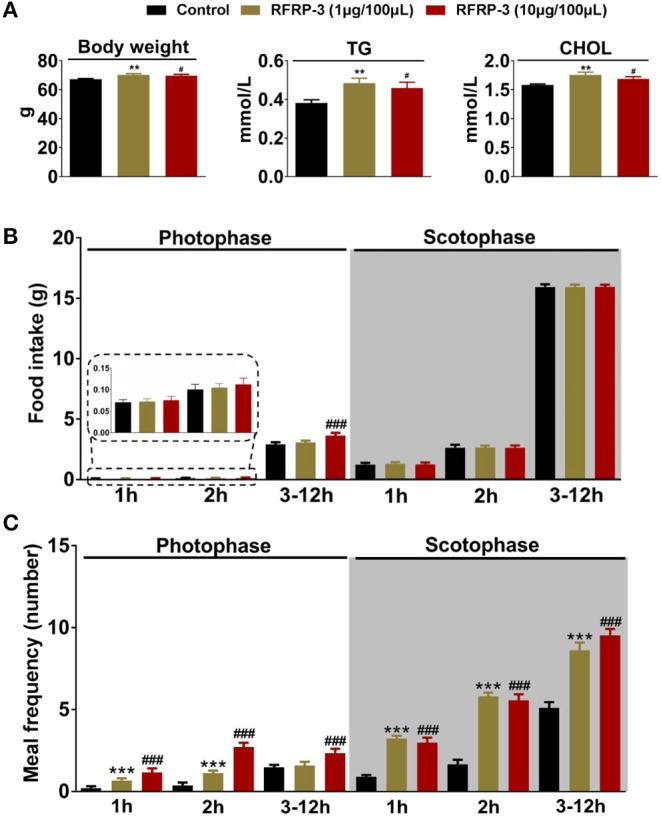
Intraperitoneally injected RFRP-3 increases rat body mass and photophase food intake and alters the meal microstructure. **(A)** The body mass parameters of rats intraperitoneally injected with different chronic doses of RFRP-3 for 14 d. **(B,C)** The food intake **(B)** and meal frequency **(C)** were monitored over 14 d during different periods of the photophase and scotophase post-RFRP-3 injection in the *ad libitum-*fed rats. *n* = 10/group. The data are presented as the means ± SEM. **p < 0.01 and ***p < 0.001 RFRP-3 1 μg/100 μL vs. vehicle; ^#^*p* < 0.05 and ^###^*p* < 0.001 RFRP-3 10 μg/100 μL vs. vehicle.

After 14 d of RFRP-3 administration, the body weights and serum total triglyceride and cholesterol concentrations were significantly increased in the low- and high-dose groups compared with the control group (Figure 1A). These data showed no significant difference between males and females (Supplementary Figure 1).

The effect of intraperitoneally injected RFRP-3 on the structure of the first meal in rats fasted for 8 h was initially investigated in our food intake analysis. As shown in Table 2, the two different doses of RFRP-3 significantly increased the food intake, duration and eating rate of the first meal compared to the vehicle-treated group.

**Table 2:** Effect of RFRP-3 injected intraperitoneally on the structure of first meal in the fasting rats.

**Parameters**	**Control (0 μg/100 μL)**	**RFRP-3 (1 μg/100 μL)**	**RFRP-3 (10 μg/100 μL)**
Food intake 1st meal (g)	2.50 ± 0.11	4.41 ± 0.52**	4.57 ± 0.24^###^
Duration 1st meal (min)	16.95 ± 0.26	20.46 ± 0.36***	21.49 ± 0.50^###^
Eating rate 1st meal (mg/s)	2.46 ± 0.12	3.59 ± 0.38*	3.54 ± 0.29^##^

Data are mean ± SEM; n = 10 rats/group. *p < 0.05, **p < 0.01, and ***p < 0.001 RFRP-3 1 μg/100 μL vs. vehicle; ^##^p < 0.01 and ^###^p < 0.001 RFRP-3 10 μg/100 μL vs. vehicle.

The food intake and meal microstructure were subsequently monitored over 14 d during the photophase and scotophase post-RFRP-3 injection in the *ad libitum-*fed rats (Table 3). Notably, rats that were intraperitoneally injected with a high dose of RFRP-3 exhibited significantly increased food intake during the photophase, whereas no change was observed during the scotophase post-RFRP-3 injection. Furthermore, the values obtained for the meal microstructure parameters showed that intraperitoneally injected RFRP-3 significantly increased the meal frequency and time spent at meals, whereas the meal size, eating rate, meal duration, intermeal interval and satiety ratio were significantly reduced.

**Table 3:** Effect of RFRP-3 injected intraperitoneally on meal microstructure in the *ad libitum* fed rats.

**Parameters**	**Photophase**			**Scotophase**		
	**Control** **(0 μg/100 μL)**	**RFRP-3** **(1 μg/100 μL)**	**RFRP-3** **(10 μg/100 μL)**	**Control** **(0 μg/100 μL)**	**RFRP-3** **(1 μg/100 μL)**	**RFRP-3** **(10 μg/100 μL)**
Food intake (g)	3.09 ± 0.07	3.24 ± 0.05	3.82 ± 0.09^###^	19.76 ± 0.18	19.82 ± 0.15	19.79 ± 0.15
Meal frequency (number)	2.03 ± 0.10	3.38 ± 0.07^***^	6.21 ± 0.13^###^	7.68 ± 0.17	17.71 ± 0.17^***^	18.13 ± 0.17^###^
Time spent in meals (min)	32.20 ± 1.07	56.30 ± 1.61^***^	73.66 ± 1.64^###^	123.99 ± 2.71	200.32 ± 3.91^***^	231.28 ± 5.32^###^
Meal size (g/meal)	1.56 ± 0.09	0.97 ± 0.03^***^	0.62 ± 0.02^###^	2.58 ± 0.06	1.12 ± 0.01^***^	1.09 ± 0.02^###^
Eating rate (mg/s)	1.61 ± 0.03	0.96 ± 0.02^***^	0.87 ± 0.01^###^	2.66 ± 0.04	1.65 ± 0.02^***^	1.43 ± 0.02^###^
Meal duration (min/meal)	16.20 ± 0.89	16.78 ± 0.65	11.93 ± 0.40^###^	16.20 ± 0.46	11.32 ± 0.24^***^	12.78 ± 0.37^###^
Inter-meal interval (min)	348.04 ± 20.80	197.63 ± 4.44^***^	104.54 ± 2.10^###^	77.96 ± 1.82	29.37 ± 0.36^***^	26.96 ± 0.26^###^
Satiety ratio (min/g food eaten)	223.42 ± 4.97	205.31 ± 3.91^*^	170 ± 4.12^###^	30.20 ± 0.40	26.25 ± 0.39 ^***^	24.73 ± 0.45^###^

*Data are mean ± SEM; n = 10 rats/group*.

* p < 0.05 and

*** p < 0.001 RFRP-3 1 μg/100 μL vs. vehicle;

### *p < 0.001 RFRP-3 10 μg/100 μL vs. vehicle*.

The above results indicated that light appears to play a crucial role in the RFRP-3-mediated increase in food intake in rats. Thus, the effect of intraperitoneally injected RFRP-3 on the food intake and meal frequency of rats was further assessed during different periods of the photophase and scotophase. As shown in Figures 1B,C, only the high dose of RFRP-3 significantly increased cumulative food intake during the 3-to 12 h period of the photophase, whereas no differences in food intake were observed during the 1st and 2nd h of the photophase post RFRP-3 injection compared to the vehicle-treated rats. Although rats preferentially eat more than 80% of their daily intake during the scotophase, either dose of RFRP-3 during any period of the scotophase did not result in a significant difference in food intake compared to the controls. However, the rats injected with the two different doses of RFRP-3 showed significantly increased meal frequency during the 2nd h and 3 to 12 h period of the photophase and scotophase, whereas no change was observed during the 1st h of the photophase or scotophase post-RFRP-3 injection.

### Effect of Intraperitoneally Injected RFRP-3 on Glucose Homeostasis and Glucometabolism-Related Hormone Concentrations

**Figure 2:**
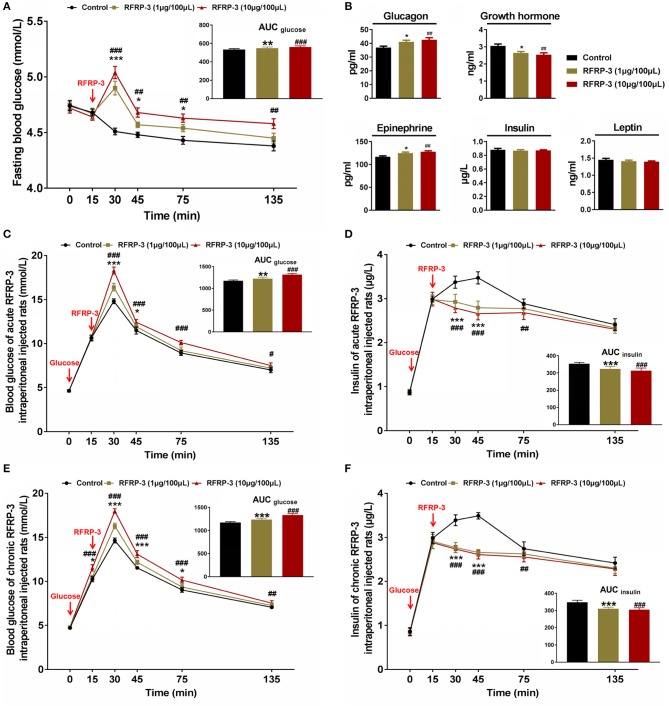
Effects of intraperitoneally injected RFRP-3 on glucose homeostasis and glucometabolism-related hormone concentrations. **(A)** The fasting blood glucose levels of rats were measured at different time points after intraperitoneally injecting different doses of RFRP-3 or vehicle into rats that had fasted for 8 h. The upper panel shows the total area under the curve (AUC) for fasting blood glucose after RFRP-3 or vehicle injection from 0 to 120 min. **(B)** The concentrations of glucagon, epinephrine, growth hormone, leptin and insulin were measured 15 min after intraperitoneally injecting different doses of RFRP-3 or vehicle into rats that had fasted for 8 h. **(C,D)** For the intraperitoneal glucose tolerance test, blood glucose **(C)** and insulin **(D)** concentrations were measured in *ad libitum-*fed rats that had been administered different acute doses of RFRP-3 or vehicle. The upper panel shows the total AUC values for blood glucose or insulin after the administration of different doses of RFRP-3 or vehicle from 0 to 120 min. **(E,F)** The blood glucose **(E)** and insulin **(F)** concentrations in the intraperitoneal glucose tolerance test were measured in *ad libitum-*fed rats administered different chronic doses of RFRP-3 or vehicle. The upper panel shows the total AUC for blood glucose after the administration of different doses of RFRP-3 or vehicle from 0 to 120 min. *n* = 10/group. The data are presented as the means ± SEM. *p < 0.05, **p < 0.01, and ***p < 0.001 RFRP-3 1 μg/100 μL vs. vehicle; ^#^*p* < 0.05, ^##^*p* < 0.01, and ^###^*p* < 0.001 RFRP-3 10 μg/100 μL vs. vehicle.

The effect of intraperitoneally injected RFRP-3 on fasting blood glucose levels was first evaluated at different time points in rats fasted for 8 h. As shown in Figure 2A, the fasting blood glucose levels dramatically increased in a dose-dependent manner for 15 min post-RFRP-3 injection and then gradually decreased from 30 to 125 min after RFRP-3 injection, whereas the blood glucose levels of the vehicle-treated rats showed a slight decrease over the 135 min study period. Compared to the vehicle-treated rats, the AUC_fasting_ _blood glucose_ levels were significantly augmented in the two rat groups injected with high and low doses of RFRP-3. Since RFRP-3 markedly increased fasting blood glucose levels, which peaked at 15 min post injection, the concentrations of glucometabolism-related hormones were further evaluated at this time point (Figure 2B). The results showed that the levels of glucagon and epinephrine were significantly increased, whereas that of growth hormone was significantly decreased in a dose-dependent manner. However, we did not observe differences in the concentrations of leptin and insulin between the RFRP-3- and vehicle-injected rats.

To investigate the effects of intraperitoneally injected RFRP-3 on glucose elimination and glucose-stimulated insulin secretion, intraperitoneal glucose tolerance tests were performed for *ad libitum-*fed rats administered acute or chronic doses of RFRP-3. As shown in Figures 2C,E, the blood glucose concentrations were significantly higher in the RFRP-3-treated rats than in the vehicle-injected rats as shown by the area under the curve values, although the glucose tolerance curves appeared slightly different between the rats injected with acute and chronic doses of RFRP-3. Notably, although an almost 100% increase in the glycemic response was observed 15 min after exogenous glucose administration in all groups, the blood glucose levels in chronic RFRP-3-injected rats showed significant increases compared with the control rats, whereas no difference was observed between the rats administered an acute dose of RFRP-3 and the control rats. In addition, despite the blood glucose levels peaking at 15 min and then gradually decreasing from 30 to 120 min after RFRP-3 injection in all groups, RFRP-3 induced significant hyperglycemia throughout the duration of the test in rats treated with high acute and chronic doses of RFRP-3. In addition, significantly higher glucose levels were detected 30–45 min after the administration of low acute or chronic doses of RFRP-3 compared with the control. Furthermore, the glucose-stimulated insulin secretion corresponded with blood glucose levels in the rats administered both acute and chronic doses of RFRP-3. As shown in Figures 2D,F, insulin secretion significantly increased 15 min after exogenous glucose administration in all groups, but subsequently, the rats administered all assayed doses of RFRP-3 exhibited significantly blunted glucose-stimulated insulin secretion that lasted for 1 h in the rats administered both acute and chronic doses of RFRP-3. A similar result was observed for the reduced AUC_insulin_. These data showed no significant difference between males and females (Supplementary Figure 2). Taken together, these results indicate that RFRP-3 inhibited the exogenous glucose elimination and insulin response to glucose, which was most significant for the rats administered a high chronic dose of RFRP-3 compared with other treatments.

### Intraperitoneally Injected RFRP-3 Increases Insulin Resistance

**Figure 3:**
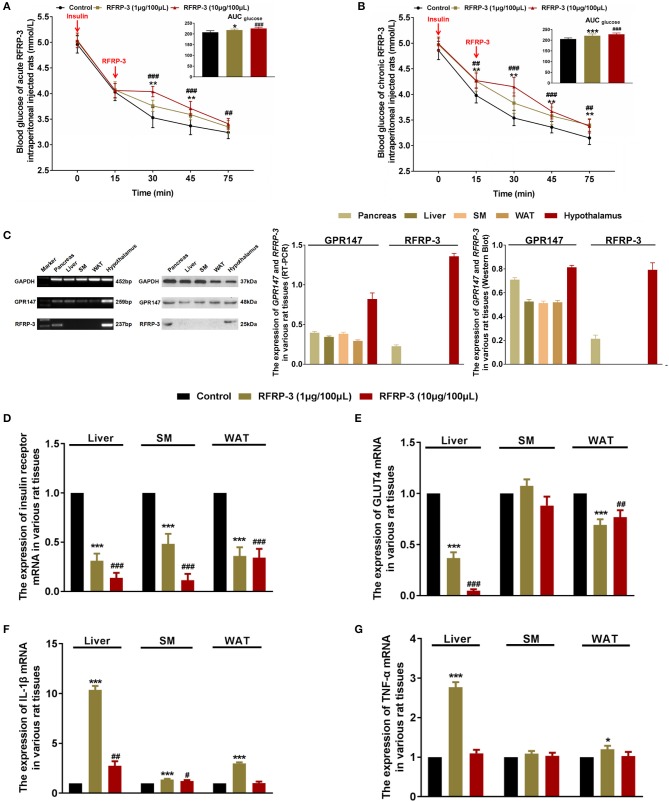
Intraperitoneally injected RFRP-3 increases insulin resistance. **(A,B)** The blood glucose levels during the insulin tolerance test were measured in rats injected intraperitoneally with acute **(A)** or chronic different doses of RFRP-3 **(B)** and were compared with those of the vehicle-treated rats. The panel shows the total AUC for blood glucose after the administration of different doses of RFRP-3 or vehicle from 0 to 60 min. **(C)** The mRNA and protein expression levels for GPR147 and RFRP-3 were evaluated by qRT-PCR and Western blot analysis in the pancreas, liver, skeletal muscle, white adipose tissue and hypothalamus in rats. The hypothalamus samples were used as positive controls; GAPDH was used as an internal control. **(D,E)** The mRNA expression of insulin receptor **(D)** and GLUT4 **(E)** in the liver, skeletal muscle and white adipose tissue of rats administered different chronic doses of RFRP-3 or vehicle for 14 d. **(F,G)** The expression of IL-1β and TNF-α mRNA in the liver, skeletal muscle and white adipose tissue of rats administered different chronic doses of RFRP-3 or vehicle for 14 d. *n* = 10/group. SM, skeletal muscle; WAT, white adipose tissue. The data are shown as the means ± SEM. *p < 0.05, **p < 0.01, and ***p < 0.001 RFRP-3 1 μg/100 μL vs. vehicle; ^#^*p* < 0.05, ^##^*p* < 0.01, and ^###^*p* < 0.001 RFRP-3 10 μg/100 μL vs. vehicle.

To determine the effect of intraperitoneally injected RFRP-3 on insulin sensitivity, insulin tolerance was measured in rats administered acute and chronic doses of RFRP-3. Our results showed that both acute and chronic injection of high and low doses of RFRP-3 significantly weakened insulin-induced hypoglycemia between 15 and 30 min after insulin challenge (Figures 3A,B). Interestingly, 15 min after intraperitoneal insulin administration, the blood glucose level was already significantly higher in rats treated with chronic but not acute doses of RFRP-3 compared with the control rats. Furthermore, the effect of RFRP-3 reduced insulin-induced hypoglycemia lasted longer in rats treated with chronic doses of RFRP-3 than those treated with acute doses. These data showed no significant difference between males and females (Supplementary Figure 3). In sum, these results indicate that both acute and chronic administration of RFRP-3 reduces insulin sensitivity in rats.

As described above, rats that were chronically administered RFRP-3 exhibited notable glucose intolerance with simultaneous low insulin sensitivity. These data prompted us to further investigate whether rats chronically treated with RFRP-3 displayed a change in insulin resistance in blood glucose-regulating organs. To identify how RFRP-3 regulates target organs in the form of autocrine or paracrine effects via its receptor GPR147, we first assessed the expression levels of RFRP-3 and GPR147 in the pancreas, liver, skeletal muscle and white adipose tissue, using the hypothalamus as a positive control (Figure 3C). Our results showed that RFRP-3 was expressed only in the pancreas, whereas GPR147 was also expressed in the liver, skeletal muscle and white adipose tissue. In addition, the expression of insulin receptor and GLUT4 mRNA was detected in the liver, skeletal muscle and white adipose tissue (Figures 3D,E). In contrast, insulin receptor and GLUT4 expression levels were significantly decreased in the liver, and white adipose tissue, with decreased expression also observed for the insulin receptor in skeletal muscle in response to RFRP-3 treatment.

### Intraperitoneally Injected RFRP-3 Increases Inflammation

Since inflammation is involved in the development of insulin resistance, we assessed whether rats chronically administered RFRP-3 via intraperitoneal injection exhibited increased inflammation indices. As shown in Table 4, the concentrations of IL-1α, IL-1β, MCP-1, and TNF-α were significantly increased in the rats treated with a low dose of RFRP-3, whereas the IL-1β and TNF-α contents were considerably only increased in the high-dose group compared with the control group. No significant differences were observed between the RFRP-3 treatment and control groups for the IL-2, IL-4, IL-6, IL-10, IL-13, or IFN-γ concentrations.

**Table 4:** The concentrations of inflammatory cytokines were detected by a protein chip array in rats administered different doses of RFRP-3 or vehicle treated for 14 d.

**Parameters**	**Control (0 μg/100 μL)**	**RFRP-3 (1 μg/100 μL)**	**RFRP-3 (10 μg/100 μL)**
IL-1α (pg/ml)	44.49 ± 2.04	61.03 ± 2.57^**^	51.98 ± 2.87
IL-1β (pg/ml)	49.62 ± 2.24	126.91 ± 7.23*^**^	65.56 ± 3.69^##^
IL-2 (pg/ml)	128.55 ± 2.89	129.50 ± 3.00	122.15 ± 3.34
IL-4 (pg/ml)	3.66 ± 0.23	3.51 ± 0.18	3.59 ± 0.21
IL-6 (pg/ml)	45.69 ± 2.14	49.02 ± 1.91	52.69 ± 2.42
IL-10 (pg/ml)	176.41 ± 2.91	168.45 ± 3.90	172.42 ± 0.93
IL-13 (pg/ml)	63.43 ± 1.04	62.72 ± 1.15	62.44 ± 1.23
MCP-1 (pg/ml)	362.36 ± 28.68	494.09 ± 31.36^**^	415.37 ± 26.85
IFN-γ (pg/ml)	11.60 ± 0.54	11.76 ± 0.31	11.58 ± 0.32
TNF-α (pg/ml)	65.63 ± 1.31	116.69 ± 3.95*^**^	74.92 ± 2.78^#^

*Data are mean ± SEM; n = 10 rats/group*.

** p < 0.01 and

*** p < 0.001 RFRP-3 1 μg/100 μL vs. vehicle;

# p < 0.05 and

## *p < 0.01 RFRP-3 10 μg/100 μL vs. vehicle*.

To validate the data obtained from the above inflammation factor analysis, the expression profiles of two representative upregulated genes, IL-1β and TNF-α, were analyzed in liver, skeletal muscle and white adipose tissue samples of rats by relative quantitative real-time PCR. As shown in Figures 3F,G, IL-1β mRNA expression levels were markedly increased in all of the assayed tissues of rats administered different doses of RFRP-3, except in the white adipose tissue of rats given a high dose of RFRP-3. However, TNF-α expression levels were only significantly increased in the liver and the white adipose tissue of the rats administered a low dose of RFRP-3, as well as in the white adipose tissue of rats administered a high dose of RFRP-3. Notably, RFRP-3 had no effect on TNF-α expression in skeletal muscle. Although the expression of TNF-α appeared to be different from that of IL-1β, a low dose of RFRP-3 had a more significant effect on increasing IL-1β and TNF-α expression levels, consistent with the results of the protein chip array analysis.

### Effect of Intraperitoneally Injected RFRP-3 on AKT-GSK Signaling

**Figure 4:**
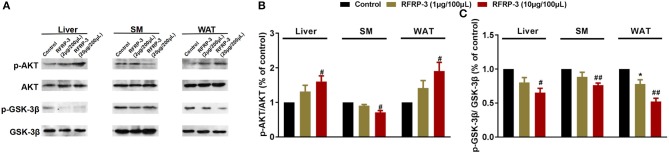
Effect of intraperitoneally injected RFRP-3 on AKT-GSK signaling. **(A)** Immunoblot of total AKT and GSK-3β, as well as phosphorylated AKT (p-AKT) and GSK-3β (p-GSK-3β), in the liver, skeletal muscle and white adipose tissue of rats administered different chronic doses of RFRP-3 or vehicle for 14 d. **(B,C)** Densitometric quantification of p-AKT and p-GSK-3β levels was performed after normalization to total AKT and GSK-3β levels as loading controls, respectively. *n* = 3 independent experiments. SM, skeletal muscle; WAT, white adipose tissue. The data are shown as the means ± SEM. *p < 0.05 RFRP-3 1 μg/100 μL vs. vehicle; ^#^*p* < 0.05 and ^##^*p* < 0.01 RFRP-3 10 μg/100 μL vs. vehicle.

To elucidate the molecular mechanism underlying RFRP-3-mediated insulin resistance, we assessed whether RFRP-3 regulates the insulin-associated AKT-GSK3-β signaling cascade (Figure 4). RFRP-3-induced phosphorylation of AKT increased in a dose-dependent manner, but only the high-dose treatment of RFRP-3 showed significantly greater AKT phosphorylation in liver and white adipose tissue. Specifically, AKT phosphorylation was significantly suppressed in skeletal muscle in rats treated with high levels of RFRP-3. In contrast, RFRP-3-induced phosphorylation of GSK3-β was significantly decreased in a dose-dependent manner.

### Colocalization of RFRP-3 and GPR147 With Insulin or Glucagon in the Pancreas

**Figure 5:**
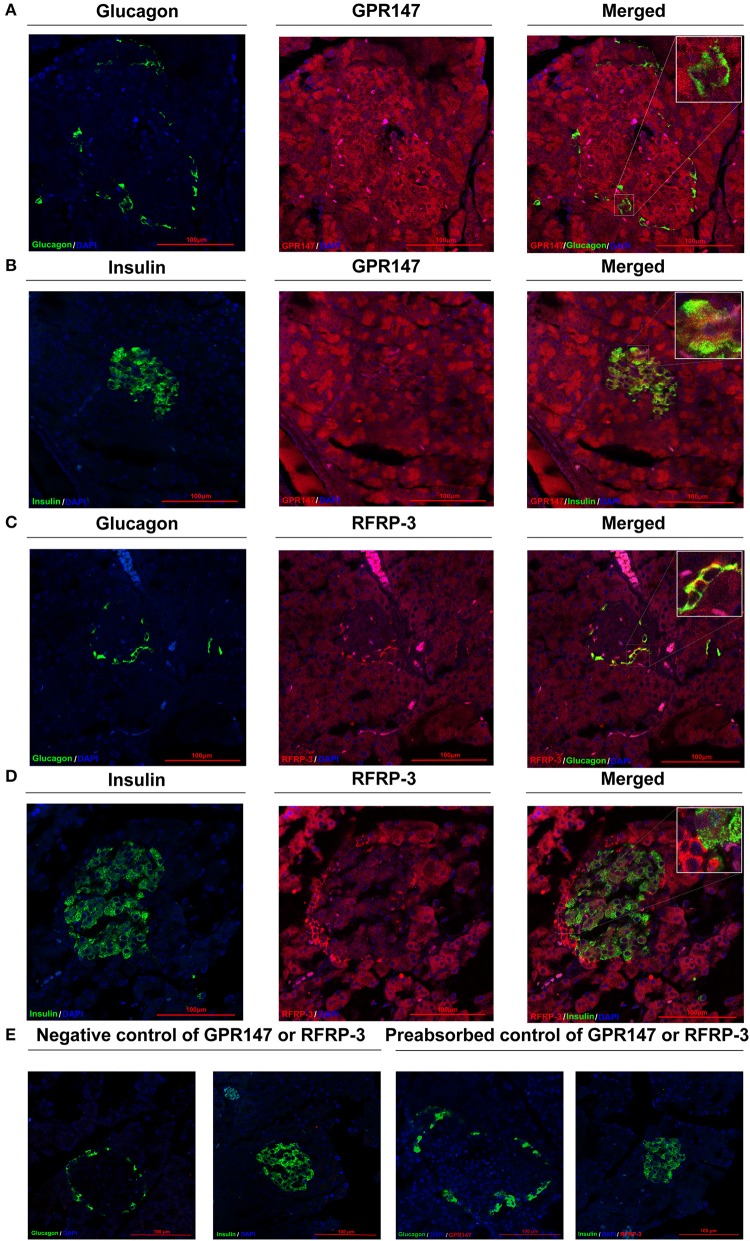
Colocalization of RFRP-3 or GPR147 with insulin or glucagon in the pancreas. **(A)** Colocalization of GPR147 (red) and glucagon (green); **(B)** GPR147 (red) and insulin (green); **(C)** RFRP-3 (red) and glucagon (green); **(D)** RFRP-3 (red) and insulin (green) in rat pancreatic islets; **(E)** Negative control and preabsorbed control of GPR147 or RFRP-3. Nuclear staining was performed with DAPI (blue). The yellow-orange color indicates colocalization. Scale bar = 100 μm, *n* = 3 independent experiments.

To elucidate the mechanism of RFRP-3 induced hypoinsulinism and hyperglucagon in rats chronically administered RFRP-3, the colocalization of RFRP-3 or GPR147 with insulin or glucagon in the pancreas was assessed by immunofluorescence double staining As shown in Figure 5, RFRP-3 and GPR147 were widely distributed in the pancreas, with mostly moderate to weak immunoreactions. Notably, some α-cells showed intense RFRP-3 immunoreactions, whereas moderate GPR147 immunoreactivity was observed around the islet and exocrine β-cells of the pancreas and weakly immunoreacted with α-cells. Thus, the confocal microscopy results revealed that RFRP-3 primarily colocalized with glucagon, whereas GPR147 primarily colocalized with insulin in the pancreatic islets.

### Intraperitoneally Injected RFRP-3 Induces Histological Changes in Pancreatic Islets

**Figure 6:**
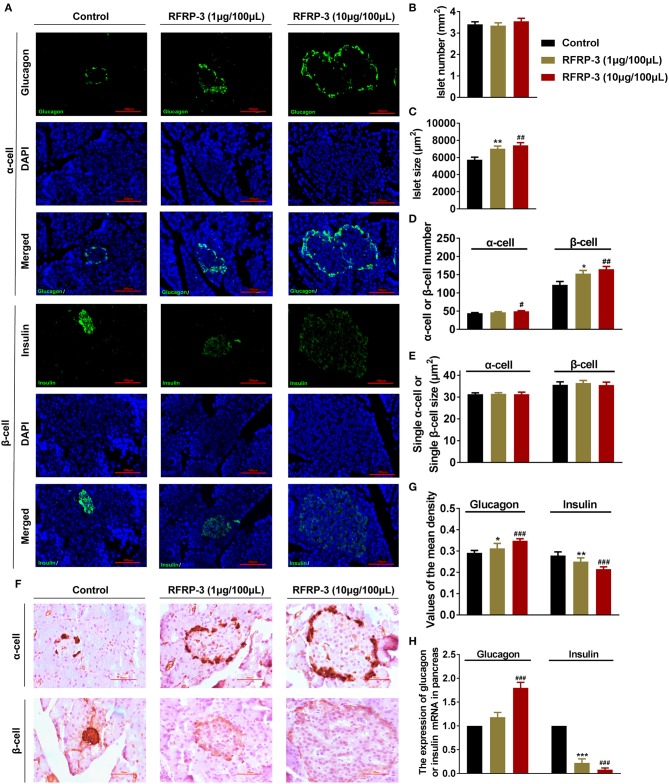
Intraperitoneally injected RFRP-3 induces histological and hormone secretion changes in pancreatic islets. **(A)** Representative immunofluorescence images of α- or β-cells (glucagon or insulin immunostaining) of pancreas sections from rats administered different chronic doses of RFRP-3 or vehicle for 14 d. **(B–E)** Statistical analysis of the number **(B)** and size **(C)** of pancreatic islets; α- or β-cell number **(D)**; and individual α- or β-cell sizes **(E)** in rats administered different chronic doses of RFRP-3 or vehicle treated for 14 d. **(F)** Representative immunohistochemistry images and **(G)** mean densitometric values for glucagon or insulin in islets of pancreatic sections from rats administered different chronic doses of RFRP-3 or vehicle for 14 d. **(H)** Glucagon- and insulin- related gene expression in the pancreases of rats administered different chronic doses of RFRP-3 or vehicle for 14 d. *n* = 3 sections/rat, 10 rats/group, scale bar = 100 μm. The data are shown as the means ± SEM. *p < 0.05, **p < 0.01 and ***p < 0.001 RFRP-3 1 μg/100 μL vs. vehicle; ^#^*p* < 0.05, ^##^*p* < 0.01 and ^###^*p* < 0.001 RFRP-3 10 μg/100 μL vs. vehicle.

To investigate whether RFRP-3 injection altered the pancreatic islet cytoarchitecture of rats, the numbers and sizes of insulin producing β-cells, glucagon producing α-cells and pancreatic islets were assessed by immunofluorescence in rats that were administered chronic doses of RFRP-3 via intraperitoneal injection. Interestingly, RFRP-3 induced the generation of significantly larger cell sizes but no change in the number of pancreatic islets was observed (Figures 6A–C). Likewise, RFRP-3 caused marked increases in the numbers of α- and β-cells, whereas RFRP-3 had no effect on the individual sizes of these cells, which resulted in a dramatic induction of pancreatic islet hyperplasia (Figures 6D,E).

To determine whether RFRP-3 triggered pancreatic islet hypertrophy and altered islet hormone secretion, the expression profiles of insulin and glucagon were further examined via immunohistochemistry and relative quantitative real-time PCR. The results showed that the expression and secretion of insulin were significantly inhibited, accompanied by significantly elevated glucagon expression and secretion resulting from chronic RFRP-3 treatment (Figures 6F–H).

## Discussion

RFRP-3 is well-known for its crucial role in the regulation of the reproductive axis (1, 18), but in light of accumulating data, it has emerged as a novel orexigenic neuropeptide that affects feeding and is potentially involved in energy metabolism (19). However, the role of RFRP-3 in meal microstructure and glucose metabolism *in vivo* has not been studied. Thus, the aim of this study was to assess the roles of RFRP-3 with respect to these research gaps.

In 2005, Tachibana et al. first revealed that intracerebroventricular (ICV) injection of GnIH stimulated the food intake of chicks (20), suggesting that this peptide functions as a novel orexigenic peptide in the brains of chicks. Subsequently, McConn et al. observed that although selection for body weight did not alter the orexigenic effect of GnIH on feeding, fasting increased GnIH mRNA expression levels in both cell lines assayed, indicating that it is an innate hunger factor (10). In addition to chicks, a similar result that RFRP-3 elevated cumulative food intake was also observed in sheep, mice and cynomolgus monkeys (21). Although RFRP-3 was identified as a novel orexigenic peptide to stimulate the food intake via ICV infusion in some species, the effect of peripheral treated RFRP-3 on appetite has never been studied before. Our current study firstly confirmed that the effect of intraperitoneally injected RFRP-3 on food intake was similar to that of ICV injection. Unfortunately, the precise mechanism by which RFRP-3 exerts orexigenic effect has been poorly understood. A potential central mechanism was suggested to be the activation of the LHA in the GnIH orexigenic response, and NPY, POMC, and MCH are also likely involved (22, 23). However, the mechanism of peripheral treated RFRP-3 seems much more complicated. To date, a large body of evidences demonstrated that the food intake was central controlled by the hypothalamus, meanwhile peripheral nutrients and hormones also provide feedback information to modulate central pathways in the brain that further influence this physiological progress (24). Despite RFRP-3 was identified in the hypothalamus and exerts multiple effects in the brain, our findings combined with previous accumulating data indicated that RFRP-3 and its receptor GPR147 were distributed in various peripheral endocrine endpoints (25, 26), such as the gastrointestinal tract, the ovary and the pancreas. Therefore, we suggested that intraperitoneally injected RFRP-3 may act on these peripheral endocrine endpoints via its receptor to modulated peripheral hormone (i.e., insulin and glucagon) which provide peripheral feedback signals to the relevant hypothalamic feedback mechanisms of food intake. Furthermore, whether peripheral RFRP-3 can enter the brain through its receptor-mediated transport crossing the blood-brain barrier like other peripheral hormones is unclear. Thus, the precise mechanism of central and peripheral RFRP-3 treatment elevated cumulative food intake is still worthy of our further study.

In addition, the results of our study showed that the effect of intraperitoneally injected RFRP-3 on food intake in rats is different during different periods of the photophase and scotophase as well as between fasting and *ad libitum-*fed rats. Different doses of RFRP-3 significantly increased the cumulative food intake of the first meal in fasting rats, but only a high dose of RFRP-3 greatly elevated the food intake during the photophase and throughout the entire day, especially during the 3 to 12 h period of the photophase in the *ad libitum-*fed rats. Interestingly, although rats preferentially eat more than 80% of their daily intake during the scotophase, no significant difference in food intake was observed in the rats that received either dose of RFRP-3 during any period of the scotophase showed compared to the controls. These findings are consistent with those of another study in rats, suggesting that exogenous RFRP-3 could not alter the natural preference of rats for nocturnal food ingestion (9) but that the light cycle and blood glucose level affect the RFRP-3-induced food intake accumulation. Although rats received intraperitoneally and ICV during photophase showed a significant increase in food intake compared to controls.

Until now, limited information is available on RFRP-3 effect on body weight dysfunction and meal microstructure. Thus, we further measured comprehensive parameters of meal microstructure and body mass in rats that were intraperitoneally injected with RFRP-3. Microstructural analyses of meal patterns showed that the RFRP-3 orexigenic effect in rats is due to increased meal frequency and time spent in meals, reduced meal size, eating rate and meal duration associated with shortened intermeal intervals. In addition, the satiety ratio was decreased after the intraperitoneal injection of RFRP-3. These data suggested that exogenous activation of RFRP-3 pathways impairs the satiety value of food ingested during spontaneous nocturnal feeding. Similarly, the results of the body mass investigation showed that the body weights and the concentrations of serum total triglyceride and cholesterol were notably increased in the RFRP-3-treated rats, which was supported and corresponded with the results of our food intake and meal microstructure analysis. Our results agreed with those of a previous study in mice, which demonstrated that RFRP-3 treatment *in vivo* showed increased food intake and increased uptake of triglycerides in adipose tissue, resulting in increased body mass (27). In summary, intraperitoneally injected RFRP-3 increased food intake by causing changes in meal microstructure, as supported by the observed reduction in satiety and as influenced by the light cycle and blood glucose levels. Furthermore, RFRP-3-triggered food intake accumulation causes metabolic syndrome, featuring body mass increased and dyslipidemia.

Increased food intake and body mass are closely related to abnormal glucose metabolism (28, 29). Therefore, we hypothesized that RFRP-3 may be involved in glucose homeostasis. To test this hypothesis, we evaluated the effect of intraperitoneally injected RFRP-3 on blood glucose levels and the concentrations of glucometabolism-related hormones in fasting rats, as well as glucose elimination and glucose-stimulated insulin secretion in *ad libitum-*fed rats administered acute and chronic doses of RFRP-3. We were pleasantly surprised to observe that intraperitoneally injected RFRP-3 could instantly and markedly elevate blood glucose levels within 15 min, which lasted over 120 min in fasting rats. Similar results were also observed in an intraperitoneal glucose tolerance test of *ad libitum* fed rats administered acute and chronic doses of RFRP-3. Notably, the blood glucose levels were significantly increased after exogenous glucose administration in rats administered a chronic dose of RFRP-3 for 14 d, even in the absence of the last RFRP-3 injection. This is the first evidence indicating that RFRP-3 is involved in glucose metabolism, suggesting that intraperitoneally injected RFRP-3 not only induced hyperglycemia but also blunted the sensitivity of rats to exogenous glucose administration and decreased the glucose elimination response, with the results of the long-term chronic RFRP-3 treatment particularly reinforcing these effects. Interestingly, RFRP-3 markedly increased fasting blood glucose levels, which peaked at 15 min post injection, which was accompanied by increased glucagon and epinephrine levels and reduced growth hormone levels, although no influence on insulin and leptin secretion was observed. However, the RFRP-3 treatment significantly inhibited glucose-stimulated insulin secretion in *ad libitum-*fed rats administered acute or chronic doses of RFRP-3. In addition, Johnson et al. observed that ICV RFRP-3 increased growth hormone levels in rats (9). These discrepancies in insulin and growth hormone secretion may be due to the differences in the blood glucose levels between fasting and *ad libitum-*fed rats. Previous studies have confirmed that adrenaline stimulates glucagon release, which subsequently stimulates increased blood glucose levels (30, 31). Furthermore, emerging evidence has revealed that glucagon secretion and adrenergic mechanisms do not typically play an essential role in this process but are crucial to the recovery from hypoglycemia when glucagon secretion is impaired (32). Thus, we hypothesized that RFRP-3 plays a crucial role in the up regulation of blood glucose levels, primarily through inhibition of insulin production and the promotion of glucagon secretion. The possible reason for the inhibition of insulin secretion may be because RFRP-3 exert its effect through Gαi and blockade of the AC-cAMP-PKA signaling pathway (33), resulting decreased insulin secretion (34).

Based on the above research, we were prompted to investigate the precise mechanism of RFRP-3 involved in blood glucose homeostasis. In our previous investigations and those of other studies, RFRP-3 and its receptor GPR147 have been reported to be distributed not only in the central nervous system but also within numerous peripheral organs (4, 14, 25). In this study, we further confirmed that RFPR-3 and GPR147 were both expressed at the mRNA and protein levels in the rat pancreas. In addition, the immunofluorescence double-staining data revealed that RFRP-3 was primarily colocalized with glucagon, whereas GPR147 primarily colocalized with insulin in pancreatic islets. Moreover, previous studies have confirmed that G protein-coupled receptors (GPCRs) play an indispensable role in regulating the function of pancreatic β-cells and the release of insulin (35, 36). These results suggested that RFRP-3 may function in an autocrine and/or paracrine manner at the pancreatic β-cells through GPR147 to inhibit insulin production in rats. However, RFRP-3 seems to affected glucagon secretion through other mechanism because weakly GPR147 immunoreactivity was observed in the pancreatic α-cells. Previous studies have confirmed that epinephrine promoted glucagon secretion through β-adrenergic receptor in α-cells (31). Our research happened to found that RFRP-3 markedly increased fasting blood glucose levels, which was accompanied by increased glucagon and epinephrine levels. Therefore, we suggested that RFRP-3 stimulated glucagon secretion by an indirect effect on the pancreatic α-cells that is probably mediated by epinephrine go up. Furthermore, anatomical and histological data have demonstrated that chronic intraperitoneal RFRP-3 treatment triggered a marked increase in the numbers of α- and β-cells, dramatically promoting pancreatic islet hyperplasia. Concomitantly, the synthesis and secretion of insulin were significantly decreased, but glucagon did the opposite. A related study performed using mouse pancreatic islets and cells from alpha TC1 clone 6 showed that RFRP-3 promotes the survival of alpha cells via GPR147 (26). Based on these findings, we hypothesized that the possible reason for the observed pancreatic islet hyperplasia was that RFRP-3-stimulated α-cell proliferation resulted in increased glucagon production, causing β-cells to compensate to maintain sufficient insulin to promote glucose homeostasis. Nevertheless, we were particularly interested in the RFRP-3-induced β-cell dysfunction. Previous studies have demonstrated that β-cell dysfunction, including insulin secretion and compensatory changes in β-cell mass, is essential for the development of the diabetes phenotype, suggesting that the proliferation of β-cells occurs before the onset of overt hyperglycemia (37). In addition, in type 2 diabetes, there is an overall reduction in the insulin secretion rate, as the β-cells can no longer secrete sufficient insulin to maintain normal blood glucose levels (38). Thus, we speculated that RFRP-3 may be a key circulating endocrine factor involved in the process of diabetes.

We also attempted to decipher the molecular mechanism underlying the RFRP-3-mediated insulin resistance and inflammation response in insulin-stimulated glucose disposal tissues. The results of our insulin tolerance test indicated that RFRP-3 reduced insulin sensitivity in rats injected with acute and chronic injection doses of RFRP-3. This conclusion was further supported by the reduced expression observed for insulin receptor and GLUT4, as well as the suppression of GSK-3β signaling in the liver and white adipose tissue. The results of a previous study in mice were consistent with our results, where RFRP-3 was also observed to promote a significant decrease in the expression of insulin receptor, and a low dose (20 ng/day) of RFRP-3 resulted in a significant decrease in the expression of GLUT4 in adipose tissue (27). Furthermore, the results of our inflammation protein chip array analysis confirmed that the concentrations of IL-1α, IL-1β, MCP-1, and TNF-α were significantly increased in the serum of rats receiving chronic intraperitoneal administration of RFRP-3. These results were supported by the observed expression of IL-1β and TNF-α in the liver and white adipose tissue of rats. However, the expression of GLUT4 and AKT phosphorylation in skeletal muscle appeared to be different with respect to the liver and white adipose tissue, indicating that tissues other than muscle appear to be more involved in RFRP-3-mediated insulin-regulated glucose disposal. As noted above, these changes in the decreased uptake of glucose, suppression of insulin signaling and increased inflammatory response may be responsible for RFRP-3-induced insulin resistance. Interestingly, accumulating data indicate that insulin resistance is a common feature of type 2 diabetes, obesity, and hyperlipidemia (39). Moreover, the pancreatic islet compensatory response to insulin resistance is a recognized feature in obesity and type 2 diabetes (40). Therefore, our findings and the results of previous studies strongly suggest that RFRP-3 is a key regulator involved in the occurrence and development of diabetes.

Based on all of our findings, we summed up that acute and chronic intraperitoneally injected RFRP-3 both elevated blood glucose levels, the possible mechanism is peripheral RFRP-3 directly acts on pancreatic islets β-cell though its receptor GPR147 to inhibit the insulin secretion. Insulin is known as a major regulator of blood glucose levels and actions of insulin in the hypothalamic feedback loop of glycometabolism regulation (24, 41). However, long-term peripheral treatment with RFRP-3 could persistently decrease the circulating insulin which may not only lead to changes in the hypothalamic circuits of glucose homeostasis and morphology of pancreatic islets but also result insulin resistance and inflammatory changes in insulin-stimulated glucose disposal tissues. These maybe the long-term chronic RFRP-3 treatment mechanism and the reasons why RFRP-3 treatment chronic showed more strong effect on blunting the sensitivity of rats to exogenous glucose administration and decreasing the glucose elimination response than acute treatment.

Altogether, the results of this study demonstrated that, when injected intraperitoneally into rats, RFRP-3, the mammalian ortholog of GnIH, triggered food intake accumulation though changes in meal microstructure and caused metabolic syndrome featuring, body mass increased and dyslipidemia. Furthermore, we provided *in vivo* and morphological data demonstrating the mechanism of RFRP-3 involved in blood glucose homeostasis, suggesting that RFRP-3 causes body mass increased, hyperphagia, hyperlipidemia, hyperglycemia, glucose intolerance, hypoinsulinism, hyperglucagon and insulin resistance, as well as the pancreatic islet compensatory response, which may contribute to the occurrence and development of diabetes. Therefore, we strongly assert that RFRP-3 as a novel neuroendocrine regulator involved in blood glucose homeostasis.

## Data Availability Statement

The datasets generated during and/or analyzed during the current study are available in the Mendeley repository. https://data.mendeley.com/datasets/z5vdx6fg2t/draft?a=eb02b0c2-e986-4217-96e9-0e012b7d47bf.

## Ethics Statement

All of the experiments were performed in accordance with the guidelines of the regional Animal Ethics Committee and were approved by the Guangxi University Ethical Committee (Project ID 2019-068).

## Author Contributions

XW conceived, designed and interpreted experiments and wrote the manuscript. KH and XL designed and performed the experiments and made the figures. WH, XS, and XZ performed and participated in interpreting the mRNA and protein expression experiments. DZ performed the morphology studies. XC, JY, and JZ assisted with animal studies.

### Conflict of Interest

The authors declare that the research was conducted in the absence of any commercial or financial relationships that could be construed as a potential conflict of interest.

## Supplementary Material

Supplementary Figure 1 Intraperitoneally injected RFRP-3 increases rat body mass in male and female rats. **(A–C)** The body mass parameters of male and female rats intraperitoneally injected with different chronic doses of RFRP-3 for 14 d. *n* = 5/group. The data are presented as the means ± SEM. ^*^*p* < 0.05 and ^**^*p* < 0.01 RFRP-3 1 μg/100 μL vs. vehicle; ^#^*p* < 0.05 and ^##^*p* < 0.01 RFRP-3 10 μg/100 μL vs. vehicle.

Supplementary Figure 2 Effects of intraperitoneally injected RFRP-3 on glucose homeostasis in male and female rats. **(A,B)** The fasting blood glucose levels of male and female rats were measured at different time points after intraperitoneally injecting different doses of RFRP-3 or vehicle into rats that had fasted for 8 h. The upper panel shows the total area under the curve (AUC) for fasting blood glucose after RFRP-3 or vehicle injection from 0 to 120 min. **(C–F)** For the intraperitoneal glucose tolerance test, blood glucose **(C,D)** and insulin **(E,F)** concentrations were measured in *ad libitum*-fed male and female rats that had been administered different acute doses of RFRP-3 or vehicle. The upper panel shows the total AUC values for blood glucose or insulin after the administration of different doses of RFRP-3 or vehicle from 0 to 120 min. **(G–J)** The blood glucose **(G,H)** and insulin **(I,J)** concentrations in the intraperitoneal glucose tolerance test were measured in *ad libitum*-fed male and female rats administered different chronic doses of RFRP-3 or vehicle. The upper panel shows the total AUC for blood glucose after the administration of different doses of RFRP-3 or vehicle from 0 to 120 min. *n* = 5/group. The data are presented as the means ± SEM. ^*^*p* < 0.05, ^**^*p* < 0.01, and ^***^*p* < 0.001 RFRP-3 1 μg/100 μL vs. vehicle; ^#^*p* < 0.05, ^##^*p* < 0.01, and ^###^*p* < 0.001 RFRP-3 10 μg/100 μL vs. vehicle.

Supplementary Figure 3 Intraperitoneally injected RFRP-3 increases insulin resistance in male and female rats. **(A–D)** The blood glucose levels during the insulin tolerance test were measured in male and female rats injected intraperitoneally with acute **(A,B)** or chronic different doses of RFRP-3 **(C,D)** and were compared with those of the vehicle-treated rats. The panel shows the total AUC for blood glucose after the administration of different doses of RFRP-3 or vehicle from 0 to 60 min. *n* = 5/group. SM, skeletal muscle; WAT, white adipose tissue. The data are shown as the means ± SEM. ^*^*p* < 0.05, ^**^*p* < 0.01, and ^***^*p* < 0.001 RFRP-3 1 μg/100 μL vs. vehicle; ^#^*p* < 0.05, ^##^*p* < 0.01, and ^###^*p* < 0.001 RFRP-3 10 μg/100 μL vs. vehicle.
