# Supplementary figures and images for: RFRP-3, the Mammalian Ortholog of GnIH, Is a Novel Modulator Involved in Food Intake and Glucose Homeostasis
Source: Front Endocrinol (Lausanne). 2020 Apr 9;11:194. doi: 10.3389/fendo.2020.00194 (PMC7160250; doi:10.3389/fendo.2020.00194)

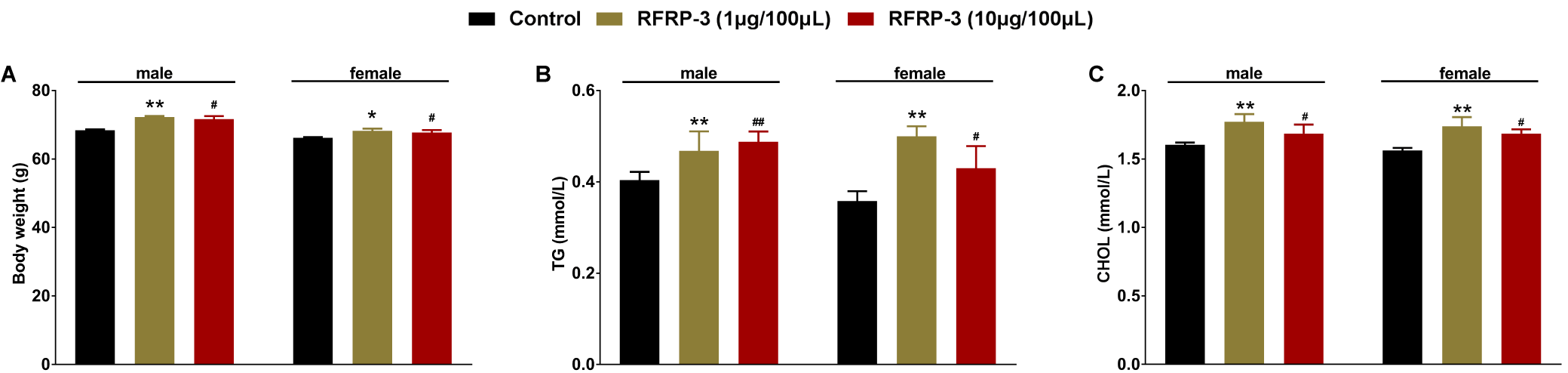

Supplement: Supplementary Figure 1 — Intraperitoneally injected RFRP-3 increases rat body mass in male and female rats. (A–C) The body mass parameters of male and female rats intraperitoneally injected with different chronic doses of RFRP-3 for 14 d. n = 5/group. The data are presented as the means ± SEM. *p < 0.05 and **p < 0.01 RFRP-3 1 μg/100 μL vs. vehicle; #p < 0.05 and ##p < 0.01 RFRP-3 10 μg/100 μL vs. vehicle. [file Image_1.tif]

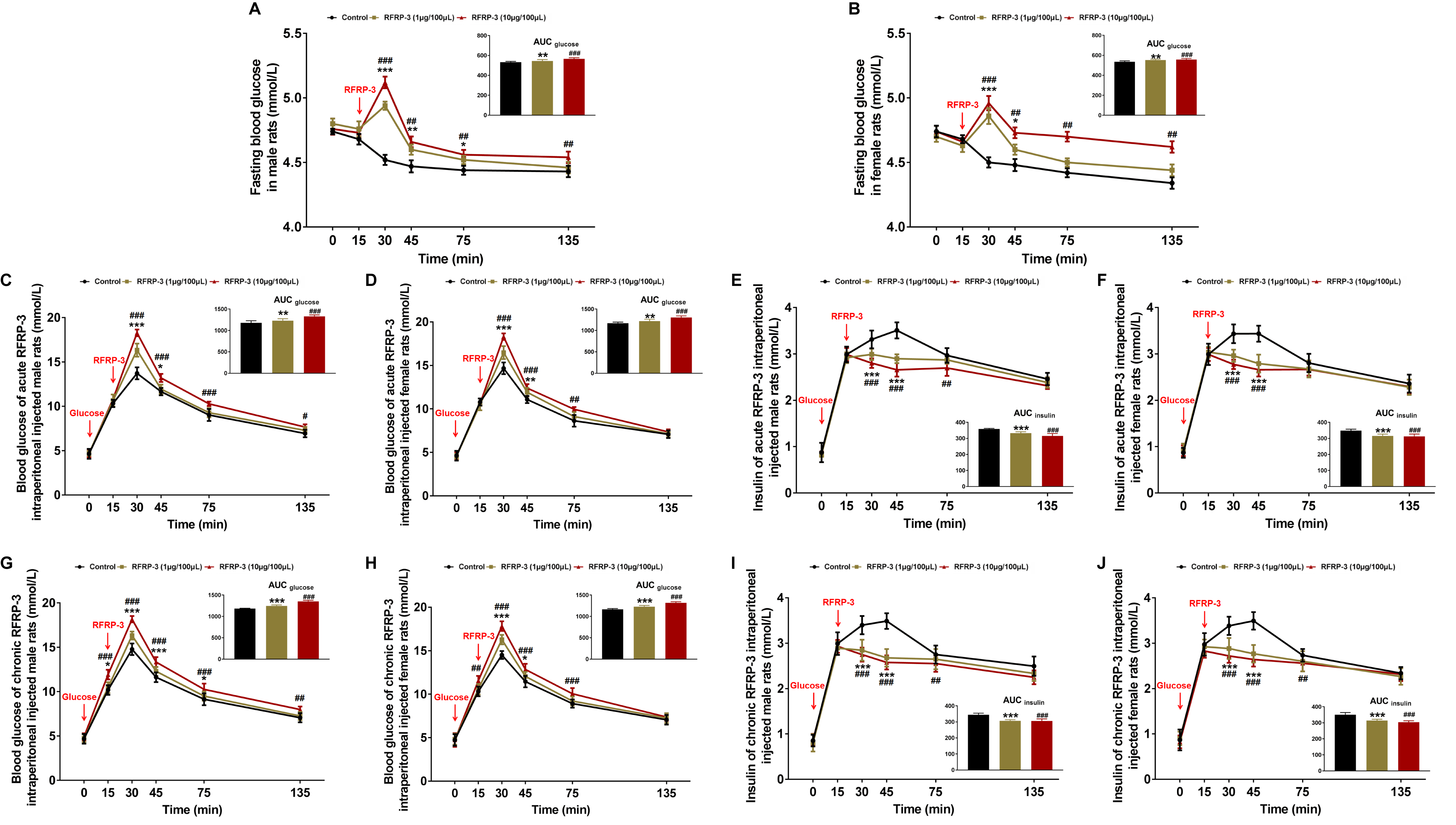

Supplement: Supplementary Figure 2 — Effects of intraperitoneally injected RFRP-3 on glucose homeostasis in male and female rats. (A,B) The fasting blood glucose levels of male and female rats were measured at different time points after intraperitoneally injecting different doses of RFRP-3 or vehicle into rats that had fasted for 8 h. The upper panel shows the total area under the curve (AUC) for fasting blood glucose after RFRP-3 or vehicle injection from 0 to 120 min. (C–F) For the intraperitoneal glucose tolerance test, blood glucose (C,D) and insulin (E,F) concentrations were measured in ad libitum-fed male and female rats that had been administered different acute doses of RFRP-3 or vehicle. The upper panel shows the total AUC values for blood glucose or insulin after the administration of different doses of RFRP-3 or vehicle from 0 to 120 min. (G–J) The blood glucose (G,H) and insulin (I,J) concentrations in the intraperitoneal glucose tolerance test were measured in ad libitum-fed male and female rats administered different chronic doses of RFRP-3 or vehicle. The upper panel shows the total AUC for blood glucose after the administration of different doses of RFRP-3 or vehicle from 0 to 120 min. n = 5/group. The data are presented as the means ± SEM. *p < 0.05, **p < 0.01, and ***p < 0.001 RFRP-3 1 μg/100 μL vs. vehicle; #p < 0.05, ##p < 0.01, and ###p < 0.001 RFRP-3 10 μg/100 μL vs. vehicle. [file Image_2.tiff]

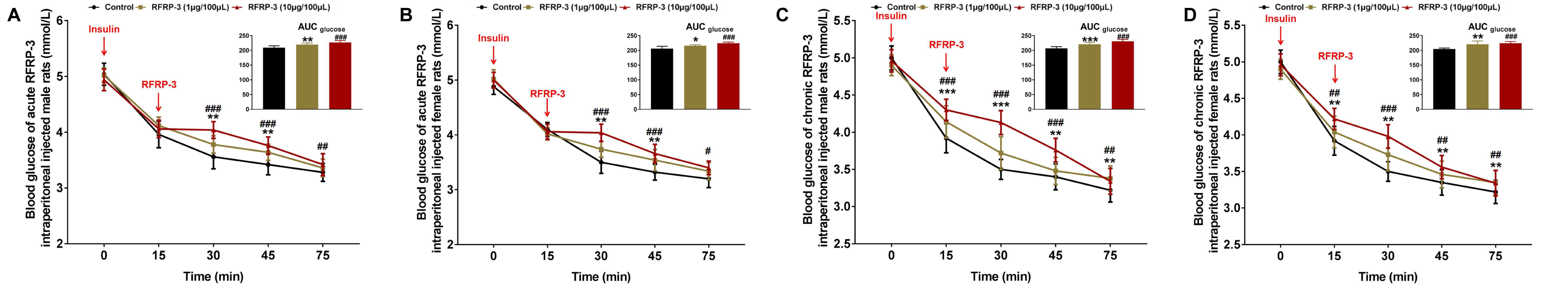

Supplement: Supplementary Figure 3 — Intraperitoneally injected RFRP-3 increases insulin resistance in male and female rats. (A–D) The blood glucose levels during the insulin tolerance test were measured in male and female rats injected intraperitoneally with acute (A,B) or chronic different doses of RFRP-3 (C,D) and were compared with those of the vehicle-treated rats. The panel shows the total AUC for blood glucose after the administration of different doses of RFRP-3 or vehicle from 0 to 60 min. n = 5/group. SM, skeletal muscle; WAT, white adipose tissue. The data are shown as the means ± SEM. *p < 0.05, **p < 0.01, and ***p < 0.001 RFRP-3 1 μg/100 μL vs. vehicle; #p < 0.05, ##p < 0.01, and ###p < 0.001 RFRP-3 10 μg/100 μL vs. vehicle. [file Image_3.tiff]
